# Genome segment ratios change during whitefly transmission of two bipartite cassava mosaic begomoviruses

**DOI:** 10.1038/s41598-023-37278-8

**Published:** 2023-06-21

**Authors:** George G. Kennedy, William Sharpee, Alana L. Jacobson, Mary Wambugu, Benard Mware, Linda Hanley-Bowdoin

**Affiliations:** 1 Department of Entomology and Plant Pathology, North Carolina State University, Raleigh, NC 27695-7630 USA; 2 Department of Entomology, Auburn University, Auburn, AL 36849 USA; 3 International Livestock Research Institute (BecA), Nairobi, Kenya; 4 Department of Plant and Microbial Biology, North Carolina State University, Raleigh, NC 27695 USA

**Keywords:** Viral transmission, Virus-host interactions, Viral vectors

## Abstract

Cassava mosaic disease is caused by a complex of whitefly-transmitted begomoviruses, which often occur in co-infections. These viruses have bipartite genomes consisting of DNA-A and DNA-B that are encapsidated into separate virions. Individual viruses exist in plants and whitefly vectors as populations comprising both genome segments, which can occur at different frequencies. Both segments are required for infection, and must be transmitted for virus spread to occur. Cassava plants infected with African cassava mosaic virus (ACMV) and/or East African cassava mosaic Cameroon virus (EACMCV), in which the ratios of DNA-A:DNA-B titers differed between plants, were used to examine how titers of the segments in a plant relate to their respective probabilities of acquisition by whiteflies and to the titers of each segment acquired and subsequently transmitted by whiteflies. The probabilities of acquiring each segment of ACMV did not reflect their relative titers in the source plant but they did for EACMCV. However, for both viruses, DNA-A:DNA-B ratios acquired by whiteflies differed from those in the source plant and the ratios transmitted by the whitefly did not differ from one – the ratio at which the highest probability of transmitting both segments is expected.

## Introduction

Multipartite viruses include ( +) and (-) sense single-stranded (ss) RNA viruses, double-stranded RNA viruses, and ssDNA viruses. They are found in 30–40% of plant virus genera and families, and 90 percent of them are transmitted by arthropod or nematode vectors^1^. Their genomes are partitioned among independent segments that are encapsidated separately into virions by the same coat protein (CP). Multipartite viruses exist in their hosts and vectors as populations comprising the different genome segments that can occur at different frequencies^2–4^.

Genome segments of the octopartite ssDNA nanovirus, *Faba bean necrotic stunt virus* (FBNSV), accumulate at different rates within infected hosts. Within a group of infected plants, there can be large variation between individual plants in the relative frequencies of the genome segments but across groups of infected plants, the median values of these relative genome segment frequencies are stable. This stable set of median values defines the setpoint genome formula of the virus, which is consistent for a given plant species, differs between plant species, and is associated with increased virus accumulation and enhanced symptoms in infected plants. It is also independent of the initial segment frequencies immediately following inoculation^5^. Genome formula changes between host species are not associated with either positive or negative selection of sequence variants, but are hypothesized to change gene expression^6^. The genome formula of FBNSV, which is aphid-transmitted in a circulative-nonpropagative manner, also differs between the vector and the host plant^7^. The discovery that FBNSV genome formula deviations from “one”, i.e., the accumulation of all genomic components in the same relative frequency, optimize host infection or transmission is counter to the long-held expectation that the genome segments occur at equal frequency and must co-infect individual cells.

Independent transmission of individual segments of multipartite viruses as a function of their relative abundance is expected to reduce the likelihood that all components needed to establish an infection are acquired and transmitted by a vector and occur in the same host cell^1,8^. However, recent research involving FBNSV revealed adaptations that mitigate this constraint^2^. These include a “multicellular way of life” in which genome segments replicate and transcribe proteins in host cells independent of the presence of other genome segments in the same cell. Movement of virally encoded mRNAs and/or proteins between cells infected with different segments allows the complete genome to be reconstituted in a plant following transmission of different subsets of the genome by different aphids^9,10^. Another adaptation allows the complete genome to be reconstituted within the vector prior to transmission. This involves accumulation and co-localization of genome segments acquired separately by an aphid vector from the same or different plants^10^. Although genome formula deviations from “one” have been documented in hosts of other multipartite viruses^4,11^, we have not identified reports for viruses other than FBNSV describing effects of differences in the relative frequencies of genome segments of multipartite viruses on the relative transmission rates of each segment by a vector and the relative frequencies of the segments that are transmitted.

The genus *Begomovirus* (family *Geminiviridae*) comprises one of the most important groups of emerging plant viruses responsible for devastating crop losses^12,13^. The growing economic importance of begomoviruses is strongly associated with global spread of their whitefly vectors^13^. Begomoviruses are characterized by circular ssDNA genomes that can be either monopartite or bipartite^14^. They are either phloem-associated or phloem-limited in plants and are transmitted exclusively by whiteflies in the cryptic species complex *Bemisia tabaci*. Transmission is circulative and nonpropagative, involving ingestion of virions during whitefly feeding in phloem tissue. The virions then pass through the whitefly’s midgut wall into the hemolymph, move through the hemolymph, and accumulate in specific cells of the primary salivary gland from which they are secreted in saliva during feeding^15^. Passage through the midgut and salivary glands involves binding of the viral CP to receptors in anterior midgut and primary salivary gland cells, and CP may be the only determinant of passage through the midgut and salivary glands^3^.

Cassava mosaic disease (CMD) is caused by a complex of 11 begomoviruses that often occur as co-infections and cause devastating losses to cassava in sub-Saharan Africa and Asia^12,16^. Cassava mosaic begomoviruses (CMBs) have bipartite genomes, consisting of DNA-A and DNA-B segments that are encapsidated into separate virions by the CP. The *AV1* gene that encodes CP resides on DNA-A along with other genes coding for proteins involved in viral replication, transcription, and interference with plant defenses. DNA-B codes for proteins involved in intra- and inter-cellular movement within plants and interference of plant defenses^17^. Both segments are required for infection, and both must be transmitted by the whitefly vector for virus spread to occur.

In our ongoing research involving the CMBs African cassava mosaic virus (ACMV) and East African cassava mosaic Cameroon virus (EACMCV), we observed substantial variation in the relative frequencies of DNA-A and DNA-B between infected cassava plants, although the median ratios for groups of plants were very close to one. Such variation among plants is consistent with that reported for FBNSV, which has a setpoint genome formula that deviates greatly from one. Given that all genome segments are encapsidated separately by the same CP and exist as populations in both plants and vectors, we conducted this study to examine how the titers of individual genomic components in plants infected with these CMBs relate to their respective likelihoods of being acquired by a vector and to the titers of each segment acquired by the vector and subsequently transmitted to an artificial diet solution or leaf discs.

## Results

Cassava plants at the 2–3 leaf stage were co-inoculated with infectious clones corresponding to ACMV DNA-A and DNA-B or EACMCV DNA-A and DNA-B to obtain singly infected plants or with all four infectious clones to obtain co-infected plants. Inoculations were conducted using low pressure biolistic bombardment to deliver the plasmid DNAs. By six to eight weeks post inoculation, the median DNA-A:DNA-B ratios in singly and co-infected plants of both viruses were very close to one but variation among plants was considerable (Table 1). The genome formula is defined by the median ratio, which for each virus is consistent between single- and co-infected plants but is slightly higher for ACMV than EACMCV.

**Table 1:** Plant to plant variation in DNA-A:DNA-B log_10_ copy number ratios of cassava plants infected with either ACMV or EACMCV or co-infected with both ACMV and EACMCV.

Infection status	Virus	n	Mean	SD	Median	Range
Single	EACMCV	16	0.95	0.212	0.87	0.61–1.27
	ACMV	20	0.93	0.087	0.94	0.62–1.05
Co-infected	EACMCV	15	0.94	0.447	0.83	0.33–2.23
	ACMV	21	1.25	1.142	0.96	0.9–6.21

Because our study focused on the relationship between viral component titers in infected plants and their acquisition and subsequent transmission by whiteflies, we chose as virus sources for whitefly transmission one plant per infection type to obtain plants that expressed a broad range of virus segment titers and ratios. We then focused on the acquisition and transmission of the DNA-A and DNA-B segments by whiteflies fed on plants having different but known viral segment titers. We measured the effects of segment titers and ratios of individual viruses in each source plant on the probabilities of acquisition and on the segment titers and ratios acquired by whiteflies from that plant. The unit of replication in the experiment is the individual whitefly exposed to infected plants of known viral segment titers and ratios. It is important to state that this experimental design does not support conclusions relating to differences between viruses or between single and co-infected plants. However, once virions are acquired by whiteflies, their subsequent transmission is expected to be affected by their composition and titers within the whitefly and independent of the effects of their abundance in the source plant. Therefore, we examined the post-acquisition effects of segment titers and ratios acquired by whiteflies and of co-acquisition of heterologous virus components by whiteflies (i.e., presence in the whitefly) on transmission.

For virus transmission by whiteflies, groups of adult *B. tabaci* SSA1-SG1 were confined on cassava plants infected with ACMV, EACMCV, or both viruses for a 48-h acquisition access period (AAP). Plants were inoculated with infectious cloned DNAs corresponding to DNA-A and DNA-B of ACMV alone, EACMCV alone, or both viruses co-precipitated in equal amounts onto gold beads for biolistic inoculation. Viral component titers were determined prior to releasing whiteflies onto the plants to initiate the AAP. Following the AAP, individual whiteflies were transferred to microcentrifuge tubes containing a sucrose solution in a Parafilm® sachet or a cassava leaf disc and held for a 48-h inoculation access period (IAP). The 48-h IAP provided sufficient time for the whiteflies to purge their gut lumen of virus-containing plant sap ingested during the AAP. Thus, it is unlikely that presence of viral components ingested during the AAP but not within midgut cells, hemolymph, or salivary glands contributed significantly to the viral titers that we subsequently measured in the whiteflies. Following the IAP, viral segment acquisition and transmission rates were estimated, and their titers in the source plants, whiteflies, sucrose, and leaf discs were measured using qPCR^18^.

Although each plant in the AAPs was inoculated with equal concentration of each genomic component, the DNA-A:DNA-B ratios differed between ACMV and EACMCV in the co-infected plant, and between the co-infected and singly infected plants. Titers of both viruses were also lower in the co-infected than the singly infected plants (Table 2). All plants were systemically infected and expressed symptoms in leaves apical to the inoculation site during the AAP.

**Table 2:** DNA-A and DNA-B titers and ratios for ACMV and EACMCV in the plants from which whiteflies acquired virus.

Infection status	Genome component	Component titer (log_10_ copy #)	DNA-A:DNA-B ratio
ACMV + EACMCV	ACMV DNA-A	4.16	6.21
	ACMV DNA-B	0.67	
	EACMCV DNA-A	3.38	0.99
	EACMCV DNA-B	3.40	
ACMV	DNA-A	27.1	0.62
	DNA-B	43.4	
EACMCV	DNA-A	18.0	0.61
	DNA-B	29.7	

### Virus acquisition by whiteflies

**Figure 1:**
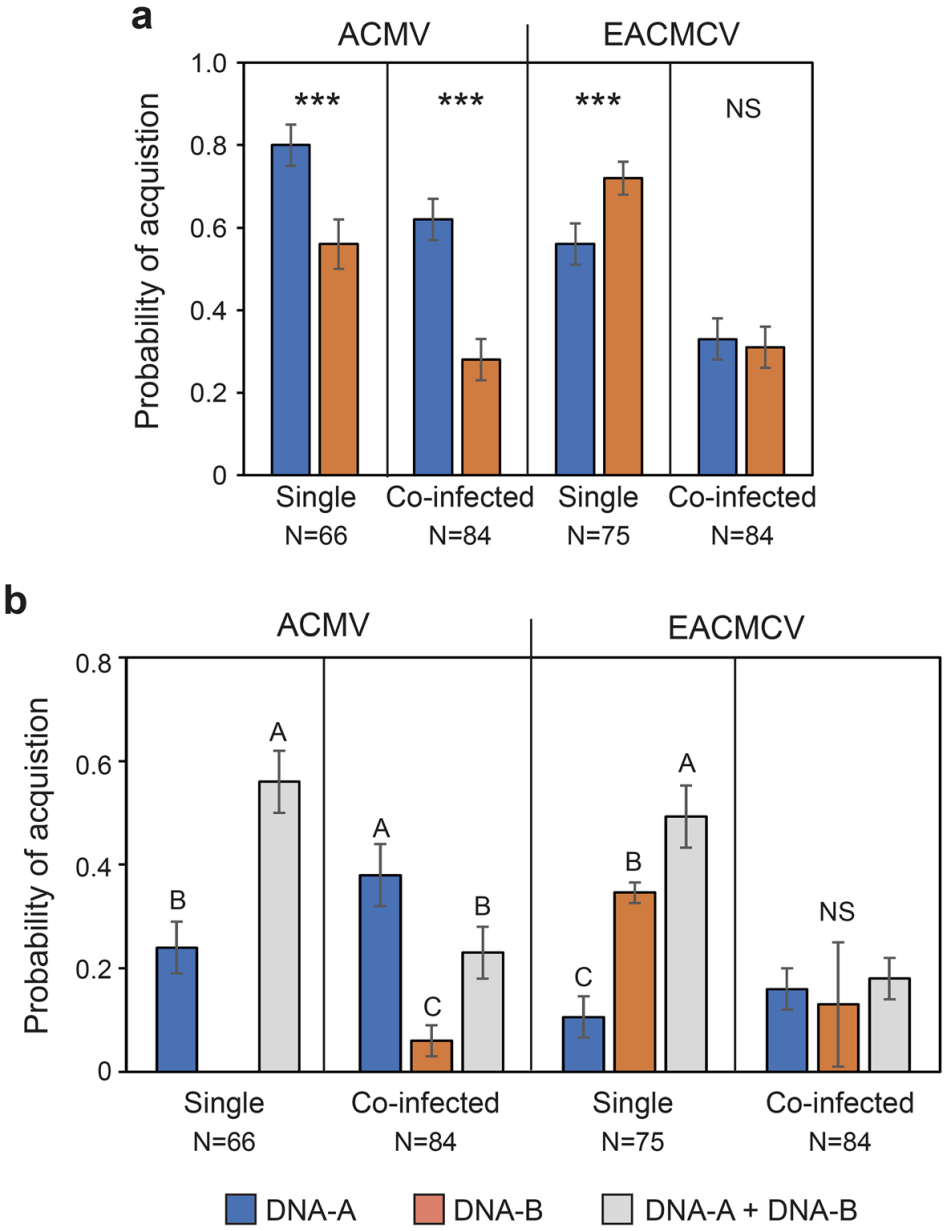
Probability of acquisition of DNA-A and DNA-B segments by *B. tabaci* SSA1- SG1 from cassava plants singly infected with ACMV or EACMCV or co-infected with both. (**a**) Probability of acquiring each segment independent of the cognate segment. *** difference between probabilities within a panel significant, alpha = 0.05. (**b**) Probability of acquiring each segment individually or with its cognate; mean separation by LSMeans at alpha = 0.05 with Bonferroni adjustment. Error bars are standard errors.

Based on the number of whiteflies that acquired DNA-A and DNA-B segments, we used logistic regression to estimate the probabilities of individual whiteflies acquiring each segment (Fig. 1A). The probabilities of acquiring each segment of ACMV did not reflect their relative titers in the source plant but did for EACMCV. The probability of acquiring ACMV DNA-A was 1.43 times greater than ACMV DNA-B from the singly infected plant in which the DNA-B titer was 1.60 times that of DNA-A. The probability of acquiring ACMV DNA-A was 2.21 times greater than DNA-B from the co-infected plant in which the ACMV DNA-A titer was 6.21 times that of DNA-B. In contrast, for EACMCV, the probabilities of acquiring DNA-A and DNA-B from the singly or co-infected plants were consistent with the relative abundance of each segment in the plants. The probability of acquiring EACMCV DNA-B was 1.28 times greater than DNA-A from the singly infected plant in which the DNA-B titer was 1.65 times greater than DNA-A. The probabilities of acquiring DNA-A and DNA-B were essentially equal (ratio = 1.06) from the co-infected plant in which the DNA-A and DNA-B titers were also equal (ratio = 0.99).

For ACMV and EACMCV, the probability of whiteflies acquiring both segments was greater than the probability of acquiring either segment alone from singly infected but not co-infected plants (Fig. 1B). For EACMCV, the probability of acquiring only DNA-B was higher than DNA-A from the singly infected plant (Fig. 1B), which is consistent with the greater abundance of DNA-B in the plant (Table 3). The probabilities of acquiring only EACMCV DNA-A, only DNA-B or both from the co-infected plant were similar, which is also consistent with the near equal abundance of each segment in the plant. However, for ACMV, whiteflies feeding on the singly infected plant never acquired DNA-B alone, despite its greater abundance in the plant. ACMV DNA-A was also acquired significantly more frequently than DNA-B from the co-infected plant in which DNA-A was 6.21 times more abundant.

**Table 3:** Comparison of DNA-A:DNA-B log titer ratios in whiteflies with the corresponding ratios in the acquisition source plants infected with both ACMV and EACMCV or only ACMV or EACMCV. Ratios in whiteflies that acquired from coinfected plants include individuals acquiring only one virus and those that also acquired at least one component of the heterologous virus.

Source plant	Virus	Source DNA-A:DNA-B ratio	Whitefly DNA-A:DNA-B ratio mean (SD; median)	t-value (df) P whitefly and source ratios not different
Co-infected	ACMV	6.21	1.33 (0.49; 1.31)	− 43.49 (18)
				< 0.0001
	EACMCV	0.99	1.13 (0.21; 1.10)	2.60 (14)
				< 0.021
Singly infected	ACMV	0.62	1.21 (0.25; 1.12)	5.43 (36)
				< 0.0001
	EACMCV	0.61	0.89 (0.09; 0.91)	19.47 (36)
				< 0.001

Using a one-sample t-test, we next tested the hypothesis that the DNA-A:DNA-B ratio of each virus acquired by whiteflies did not differ from the corresponding ratio in the plant from which the virus was acquired. In all cases, the ratios in whiteflies differed significantly from those in the plant (Table 3). Despite large differences in the ratios between the singly and co-infected plants, the ratios in the whiteflies were remarkably similar regardless of the source plant. With the exception of EACMCV acquired from the co-infected plant with DNA-A:DNA-B ratio of 0.99, the ratios in the whiteflies were closer to 1 than the corresponding ratio in the plant.

**Figure 2:**
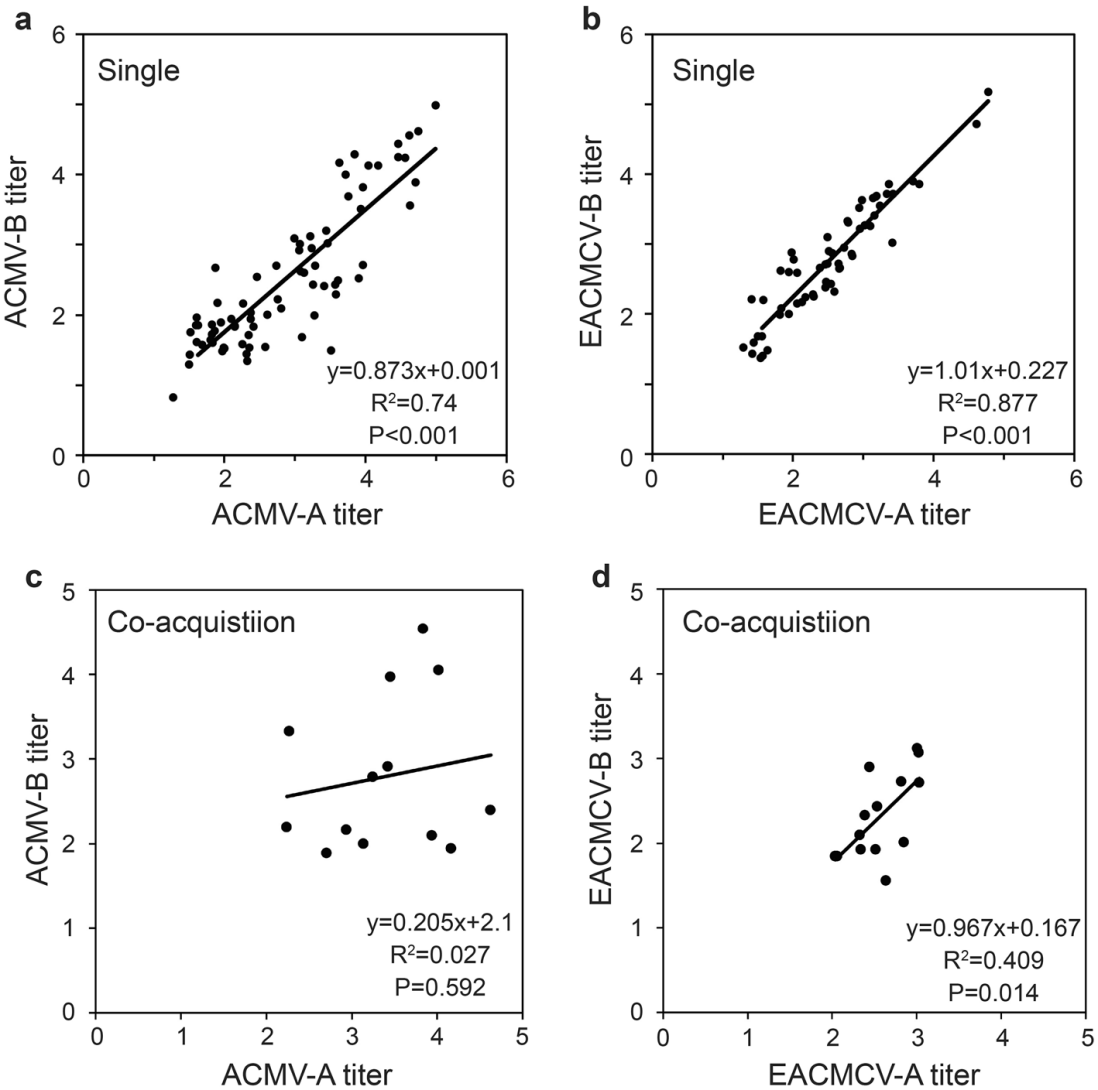
Relationship between DNA-A and DNA-B titers of ACMV and EACMCV in whiteflies that acquired both components of (**a**) only ACMV (Single); (**b**) only EACMCV (Single); (**c**) ACMV + one or both components of EACMCV (Co-acquisition); (**d**) EACMCV + one or both components of ACMV (Co-acquisition). Titers = log_10_ copy number/ng total DNA.

We next examined whether the relationship between the DNA-A and DNA-B titers of each virus were altered by the co-acquisition of one or both segments of the heterologous virus. We used linear regression to describe the relationships between the DNA-A and DNA-B titers in whiteflies that acquired both segments of only one virus regardless of whether the source plant was singly or coinfected, and in whiteflies that acquired both segments of one virus plus at least one segment of the heterologous virus (co-acquisition) from the coinfected plant. For whiteflies acquiring both segments of only one virus (ACMV or EACMCV), there is a strong positive linear relationship between the titers of DNA-A and DNA-B across a range of at least 3.5 log_10_ units (Fig. 2A and 2B). These regressions explain 74 and 88 percent of the total variation in the segment titers for ACMV and EACMCV in whiteflies, respectively. A similar, significant linear relationship between segment titers of EACMCV was observed for whiteflies that also acquired at least one ACMV segment (Fig. 2D). This relationship explained only 41 percent of the total variation. However, the slopes are similar for whiteflies acquiring EACMCV DNA-A and DNA-B only and those that also co-acquired with at least one ACMV segment (0.967 and 1.01, respectively). In contrast, the relationship between the ACMV DNA-A and DNA-B titers in whiteflies that also acquired at least one segment of EACMCV was not significant (Fig. 2C).

**Figure 3:**
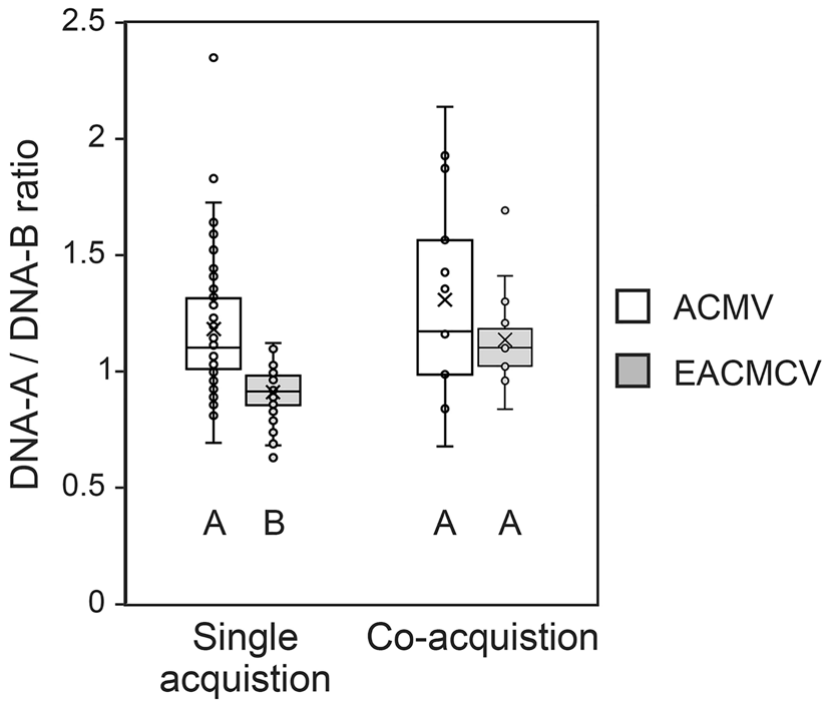
Ratios of DNA-A:DNA-B log_10_ titers in whiteflies acquiring both segments of only one virus (ACMV or EACMCV; single acquisition) or in whiteflies acquiring both segments of either ACMV or EACMCV plus at least one segment of the heterologous virus (co-acquisition). Capital letters beneath the each box plot denote mean separations. Means of box plots sharing the same letter are not significantly different (*p* =  0.05; LSMeans analysis).

We also compared the ratios of DNA-A:DNA-B titers of each virus in whiteflies from the single and co-acquisition groups (Fig. 3). In whiteflies acquiring both segments of only one virus (single acquisition), the mean DNA-A:DNA-B ratio was significantly lower and less variable for EACMCV than for ACMV. In contrast, co-acquisition of at least one segment of the heterologous virus resulted in a significant increase in the DNA-A:DNA-B ratio for EACMCV but not for ACMV. However, variation in the DNA-A:DNA-B ratios among individual co-acquiring whiteflies was increased for both viruses. The coefficient of variation (cv) of the DNA-A:DNA-B ratio for ACMV was 1.15 times greater for co-acquiring than single acquiring whiteflies (cv = 0.34 and 0.23, respectively), and for EACMCV was 1.58 times greater for co-acquiring than single acquiring whiteflies (cv = 0.19 and 0.12, respectively). The increase in the ratio for EACMCV in whiteflies that co-acquired at least one segment of ACMV likely explains the small but significant increase in the mean DNA-A:DNA-B ratio of all whiteflies that acquired both EACMCV segments from the coinfected plant, regardless of whether they co-acquired a heterologous viral segment (Table 2). Together, these results suggest that the co-acquisition of at least one heterologous viral segment disrupts the strong association between cognate DNA-A and DNA-B titers acquired by whiteflies.

### Relationship between DNA-A and DNA-B titers and transmission by whiteflies

**Figure 4:**
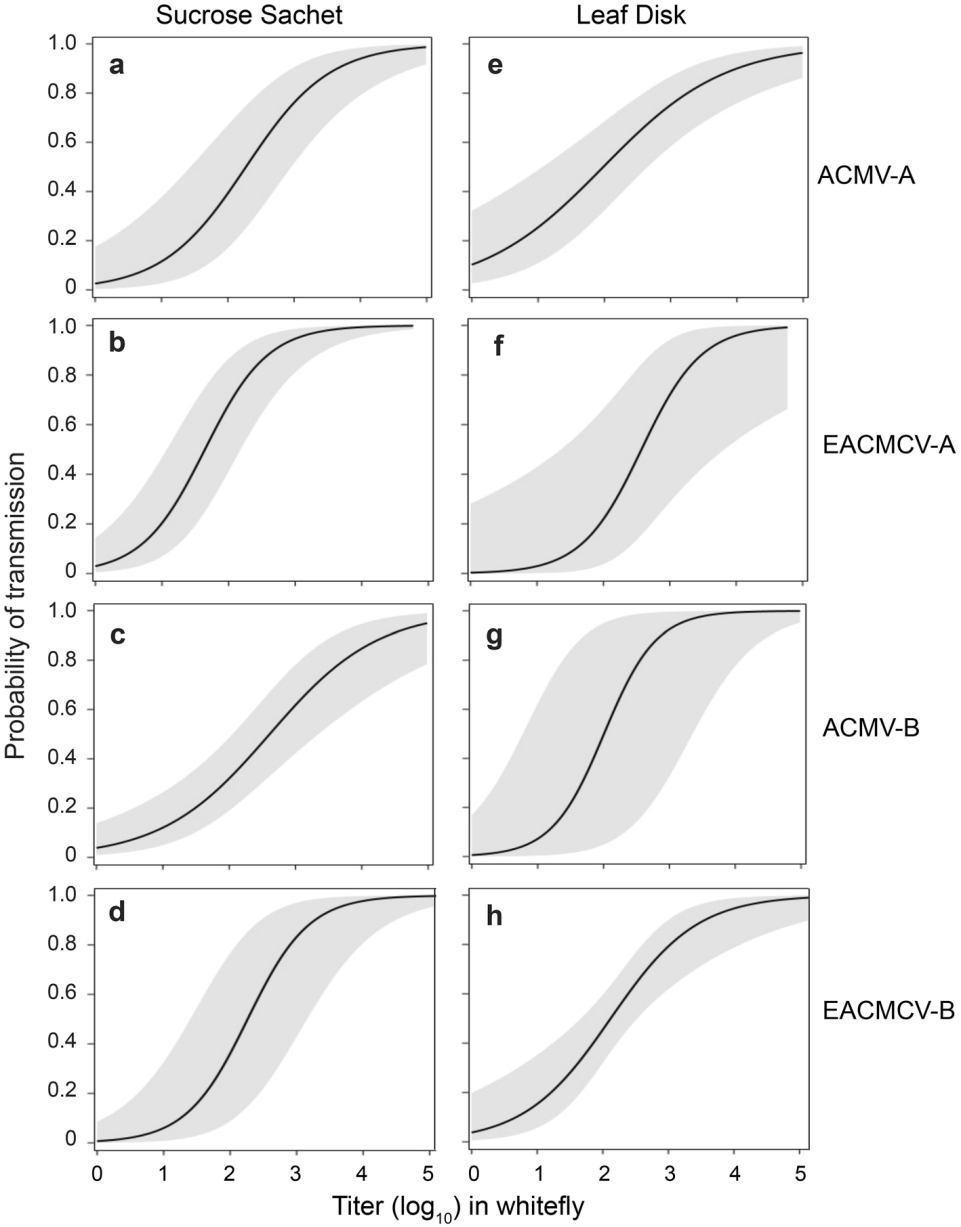
Relationship between probability of transmission of individual DNA-A or DNA-B components of ACMV and EACMCV and their respective titers (log_10_ copy number/ng total DNA) within the transmitting whiteflies. Significance levels: Sucrose sachets (**a**) *p* =  0.0003, (**b**) *p* =   < 0.0001, (**c**) *p* =  0.0002, (**d**) *p* < 0.0001; Leaf discs (**e**) *p* =  0.0007, (**f**) *p* =  0.0429, (**g**) *p* =  0.0004, (**h**) *p* =  0.0030. See supplemental Table S1 for parameter estimates. Shading represents 90% confidence intervals for the regressions.

Several studies have documented positive relationships between viral titers in their insect vectors and transmission of plant viruses^19–23^. To determine if the amounts of DNA-A and DNA-B acquired by whiteflies affect their transmission, the probabilities of their transmission to sucrose and cassava leaf discs were analyzed as a function of their respective titers in whiteflies. An initial logistic regression analysis revealed that co-acquisition of either or both heterologous viral segments by whiteflies had no effect on the probability of transmission of individual segments of either virus. Therefore, all whiteflies that acquired a given segment were included in separate analyses conducted for each segment of the two viruses. In all cases, these analyses revealed that transmission of each component into sucrose or into leaf discs was positively related to its titer in the whitefly (Fig. 4).

**Figure 5:**
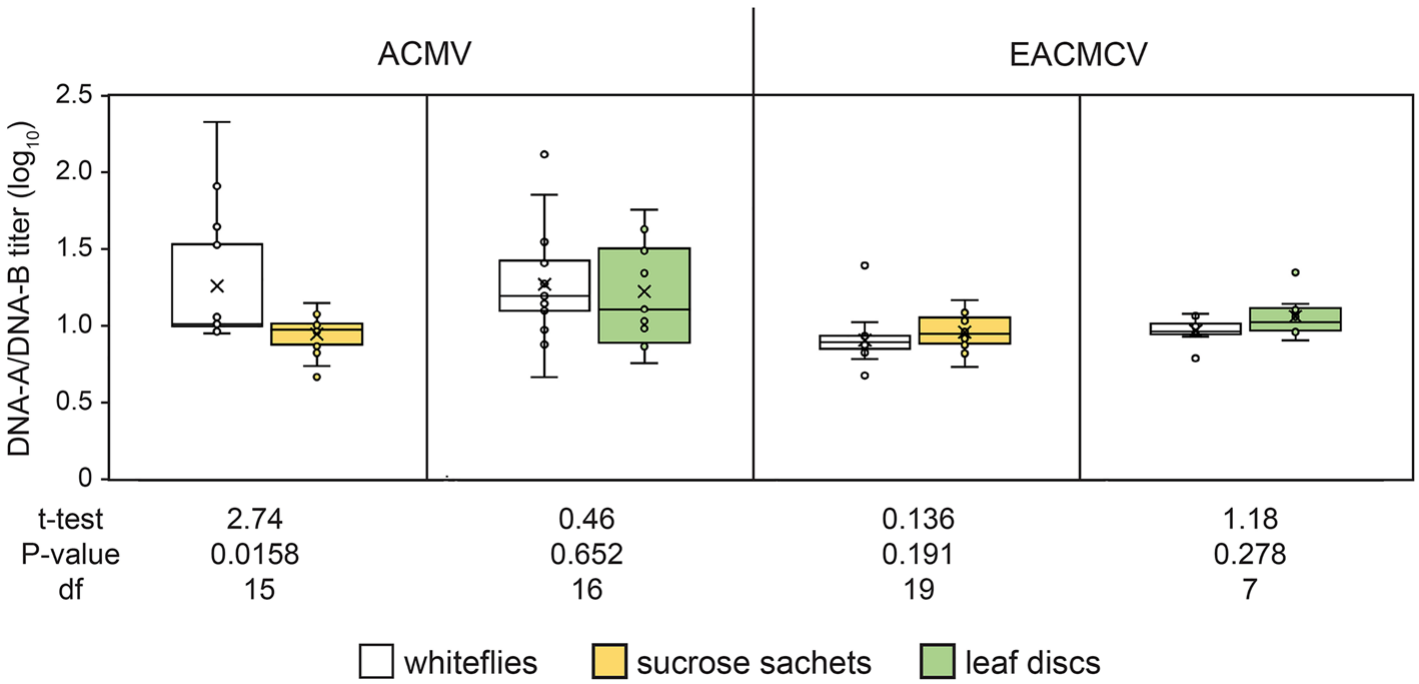
DNA-A:DNA-B log_10_ copy number ratios in transmitting whiteflies and corresponding sucrose sachets or leaf discs  for ACMV and EACMCV. P, t, and df values for paired t-tests of hypothesis that DNA-A:DNA-B ratios transmitted to sucrose or leaf discs are not different from the ratios in the transmitting whitefly.

Next, we conducted paired t-tests using the same dataset to test the hypothesis that the DNA-A:DNA-B ratios of ACMV or EACMCV transmitted to the sucrose or leaf discs did not differ from the corresponding ratios in the transmitting whiteflies (Fig. 5) and whether the ratios differed from one. The ACMV DNA-A:DNA-B titer ratio in whiteflies transmitting to sucrose sachets or leaf discs were significantly greater than one (means (cv) = 1.28 (0.34) and 1.29 (0.27), respectively; single sample t-tests: P ≤ 0.025). The ratio transmitted by whiteflies to sucrose sachets was significantly lower and less variable than in the transmitting whiteflies (*p* = 0.016). In contrast, the DNA-A:DNA-B titer ratio in the leaf discs (1.25 (0.27) did not differ significantly from that in the transmitting whiteflies (1.29 (0.27) Fig. 5). For EACMCV, there were no significant differences between the DNA-A:DNA-B ratios in the whiteflies and the sucrose sachets or leaf discs to which they transmitted. In all cases, the ratios did not differ significantly from one (Fig. 5).

Differences in these relationships between the sucrose and leaf discs likely reflect effects of virus replication and other processes (e.g., cell-to-cell movement and host defense responses) following inoculation of the leaf disc. Although it is possible that virus replication in the leaf disc during the 48 h IAP may have resulted in some virus ingestion by the whiteflies, the similarity of the DNA-A:DNA-B ratios between whiteflies that inoculated sucrose sachets and those that inoculated leaf discs does not indicate that ingestion of virus from the leaf discs affected the ratios in the whiteflies (Fig. 5).

## Discussion

Transmission of circulative, nonpropagative viruses by their vectors requires that virions penetrate the midgut, move in the hemolymph, and enter the primary salivary gland. CP plays a critical role in these processes^2,15^. Virus acquisition requires CP binding to receptors on epithelial cells in the anterior midgut, which is essential for virion passage into the hemolymph. Transmission also requires CP interactions with specific cells in the primary salivary gland for passage of virions to the salivary duct from which they are delivered in saliva to a plant during feeding. Differences among viruses in the abilities of their CP to bind midgut and/or salivary gland receptors influence transmission efficiency and vector specificity^23,24^.

For multipartite viruses, the virion population in a plant or vector consists of a mixture of genomic segments individually packaged into virions composed of the same CP. Acquisition of virions by whiteflies occurs during ingestion of phloem sap from the sieve elements. In our experimental system, DNA-A and DNA-B virions of each begomovirus occurred in different proportions, depending on the infected source plant and virus species. In the absence of processes resulting in selective acquisition and/or transmission of DNA-A and DNA-B virions by whiteflies, the likelihood of acquisition of each segment and the relative amount of each segment acquired by whiteflies is expected to reflect its abundance in infected plants. Similarly, the relative amount of each segment transmitted by the whitefly is expected to reflect its relative abundance in the vector.

Our results show differences between ACMV and EACMCV in the relationships between the relative abundance of each genome segment in the virus source plant and the likelihood that each segment is acquired from singly infected plants. For EACMCV, the probabilities that whiteflies acquire each segment, regardless of the presence of the cognate segment, were generally consistent with their relative abundance in the source plant. This was not the case for ACMV. For ACMV, the probability of acquiring DNA-A was greater than the probability of acquiring DNA-B from both the singly and co-infected plants, despite a tenfold difference in the relative abundances of DNA-A and DNA-B between these plants. Although DNA-B was more abundant than DNA-A in the singly infected plant, no whiteflies acquired only DNA-B, whereas the probability of acquiring only DNA-A was 0.24. Our finding that the probability of acquiring both the DNA-A and DNA-B segments was greater than that of acquiring either segment alone from plants singly infected with either ACMV or EACMCV is not consistent with a high dependency on titer for acquisition of individual segments.

The DNA-A:DNA-B ratios for both ACMV and EACMCV acquired by the whiteflies also differed significantly from their corresponding ratios in the source plants. The ACMV and EACMCV DNA-A:DNA-B ratios acquired by whiteflies from the singly infected plant were 1.95 times and 1.46 times higher than their corresponding ratios in the plant, respectively. This suggests that DNA-A segments are acquired more efficiently than DNA-B segments. With the exception of EACMCV acquired from the coinfected plant (DNA-A:DNA-B ratio = 0.99), the ratios in the whitefly were closer to but significantly greater than one (Table 3). However, for both viruses, the DNA-A:DNA-B ratios that the whiteflies inoculated into sucrose did not differ significantly from one. Although the EACMCV ratio change during inoculation was small (means = 0.88 to 0.98, respectively) and not significant, the ACMV ratio change was larger and significant (1.28 to 0.96, respectively) (Fig. 5). Thus, the genome segment ratios of both viruses in infected plants were altered during whitefly transmission such that the segment frequency ratio transmitted did not differ from one. The DNA-A:DNA-B ratio between the source plant and sucrose sachets changed from 6.21 to 0.96 for ACMV and from 0.61 to 0.98 for EACMCV. An expected consequence of these changes during passage through the whitefly is an increased likelihood that both genomic segments will be transmitted^25,26^.

The mechanisms mediating these changes are not known. ACMV and many other bipartite begomoviruses are phloem associated, infecting companion cells and associated parenchyma cells^27^. Therefore, it is possible that the segment ratios in the phloem sap differ from the ratios in the associated parenchyma cells. A potential difference in the segment ratios between vascular cell types and the ratios in the phloem sap ingested by the whiteflies might explain all or part of the differences between DNA-A:DNA-B ratios in plants and whiteflies. However, it cannot explain the change in the ratio of ACMV that occurred during inoculation to sucrose by a whitefly.

To our knowledge there are no reports that address the relative abundance of the DNA-A and DNA-B segments of CMBs or other bipartite begomoviruses in phloem sap versus leaf tissue. Sicard et al*.*^7^ found that the genome formula of FBNSV in phloem sap exudate of infected plants reflected the formula in the infected leaf but not the aphid vector. Changes in the FBNSV genome formula during aphid acquisition take place as the segments differentially co-locate in anterior midgut cells and accumulate in sufficient numbers to ensure that at least one copy of each genome segment is present before moving to the primary salivary gland^7,28^. Several mechanisms have been proposed for the differential accumulation of FBNSV genome segments during acquisition^7^. These include transient and differential replication of genome segments within the midgut cells, differential degradation of some segments, and differences in physicochemical properties of virions that reflect differences in the genome segments. One or more of these mechanisms could potentially explain our findings that whiteflies had a higher probability of acquiring both DNA-A and DNA-B than either segment alone, as well as the unexpectedly high likelihood of acquiring only DNA-A from plants infected with only ACMV in which DNA-B was more abundant. They could also explain the changes in DNA-A:DNA-B ratios that we observed with ACMV and EACMCV during acquisition and transmission by their whitefly vector.

Replication of the monopartite begomovirus *Tomato yellow leaf curl virus* has been demonstrated in a subset of cells in the primary salivary gland of its vector *B. tabaci* MEAM1^29^. However, there is no evidence indicating that other begomoviruses replicate in whiteflies, and studies of two bipartite begomoviruses suggest that viral replication does not occur in the vector^21,30^. Thus, transmission of most begomoviruses is considered to be circulative and non-propagative^3^, and it seems unlikely that differential replication of genome segments in the vector explains our findings.

Virus acquisition by whiteflies from the co-infected plant altered the relative probabilities of acquiring DNA-A and DNA-B of each virus, and co-acquisition of heterologous virus segments altered the relationship between the titers of each segment acquired by the whitefly. In the co-infected plant, titers of the ACMV and EACMCV DNA-A segments were relatively similar (ratio = 1.23), whereas the titers their DNA-B segments were very different (ratio = 0.197). Given these differences, it is possible that transencapsidation^31^ in which DNA segments of one virus are encapsidated by the CP of the other virus, may have affected the relative acquisition rates of DNA-A and DNA-B by whiteflies. The CPs of ACMV and EACMCV display a high level of amino acid conservation (82% identity/92% similarity) that might enable transencapsidation of a heterologous genome component. However, there are currently no epitope-specific antibodies available that can distinguish different begomovirus CPs and can be used to determine whether and at what frequency transencapsidation might occur in a co-infected plant. If transencapsidation occurred, it may have contributed to the differences in segment acquisition rates and the greater variation in the DNA-A:DNA-B ratios of both viruses acquired by whiteflies from the co-infected plant.

Although co-acquisition of heterologous viral segments altered the relative frequencies of the DNA-A and DNA-B segments of a given virus acquired by whiteflies, the likelihood of transmission of each segment was a function of its titer in the whitefly and was not affected by co-acquisition of heterologous viral segments. Therefore, an expected consequence of the convergence of the genome segment ratios on ‘one’ during passage through the whitefly is an increased likelihood that both genomic segments will be transmitted^1,2,8,26^.

Evolution of multipartite viruses is expected only when the fitness costs are outweighed by the advantages associated with multipartite genomes. Putative advantages relate to shorter genome segments, which replicate more rapidly and with greater fidelity and have longer lifespans between replication events^32^. The primary fitness cost stems from the number of virions that must enter a cell to ensure that at least one copy of each genome segment is present (referred to as multiplicity of infection – MOI). This number increases rapidly as the number of segments increases. For any given number of genome segments, the MOI is minimized when all segments occur at equal frequency (ratio = 1) and increases as the ratios diverge from one^5,32^. When all segments are equally abundant, the minimum MOI necessary to favor selection of bipartite and tripartite viruses has been estimated to be two and ca. 30, respectively, and to increase to > 1000 for viruses with ≥ 4 segments^8,32^. Nanoviruses have overcome this fitness cost by evolving a “multicellular lifestyle” and adaptations that allow reconstitution of the complete genome within the vector prior to transmission^9,10^.

The genus *Begomovirus*, which comprises both monopartite and bipartite species, is the main contributor to the overall abundance of multipartite viruses in the Virosphere^1^. Viral evolution studies suggest that begomoviruses arose from a common ancestor with a monopartite genome that gave rise to the genomes of modern monopartite begomoviruses and the DNA-A segments of bipartite begomoviruses^33^. DNA-B segments, which have a different evolutionary history than DNA-A, may have evolved from a satellite molecule captured by a monopartite progenitor of DNA-A^34,35^. Alternatively, the DNA-A and DNA-B segments may have evolved from a single ancestral DNA^36^. Either mechanism would have doubled the size of the viral genome and increased coding capacity, facilitating separation of the genes encoding movement and transmission functions. Notably, the CP of monopartite viruses is engaged in movement and transmission, while CP is involved in transmission but not in movement of bipartite viruses, which encode two movement proteins on DNA-B. This separation by bipartite begomoviruses would allow vector transmission mechanisms to evolve independently of viral movement functions^34^, potentially providing a selective advantage that could overcome in part the fitness cost of going from a one to two segment genome.

Despite the abundance of bipartite begomovirus species and an in-depth understanding of their virus-plant-vector interactions, little is known of the potential impacts of the bipartite genome structure on transmission by their whitefly vectors. We found that the mean and median DNA-A:DNA-B ratios were close to one for groups of 15–20 plants singly- or co-infected with ACMV and/or EACMCV, but the ratios varied considerably among individual plants. Although the potential fitness costs associated with multipartitism are much less for bipartite than multipartite viruses with higher numbers of genome segments, our findings show that at least two bipartite begomoviruses possess an adaptation that is expected to enhance the likelihood that all genome segments are transmitted by their vector when segment ratios in virus-infected plants deviate significantly from one. It remains for future research to determine the extent to which DNA-A:DNA-B ratios in infected plants vary among different bipartite viruses and the extent to which genome segment frequency changes occur during whitefly transmission, as well as to elucidate the mechanisms underlying changes in segment ratios during transmission. A fuller understanding of the evolution of multipartite viruses will also benefit from research to determine if the “multicellular way of life” exhibited by FBNSV is limited to multipartite viruses with high segment numbers or extends to bipartite viruses.

## Materials and methods

### Whitefly colony

Whiteflies were obtained from a colony of *Bemisia tabaci* SSA1-SG1 initiated from offspring of adults collected in July 2016 from cassava fields in Kisumu County, Kenya. The colony was maintained, and the research conducted at the BecA-ILRI Hub in Nairobi, Kenya. All founding adult whiteflies were determined to belong to the SSA1-SG1 clade based on amplification of mtCOI universal primers C1-2195 and L2-N-3014 and the procedures of Boykin and De Barro^37,38^, The colony was initially reared for at least two generations on eggplant (*Solanum melongena*), a non-host of CMBs, after which it was maintained on virus-free cassava plants (*Manihot esculenta*).

### Cassava plants and virus inoculation

Virus-free cassava plantlets (cv. Kimbandameno) in tissue culture were provided by Dr. Joseph Ndunguru at Tanzania Agricultural Research Institute and subsequently propagated in tissue culture. All plants used in the experiments were started from cuttings from these plants and grown in plastic pots maintained in whitefly-proof cages (Product: BugDorm-44545F bugdorm.com) under natural daylight (ca. 12 h) supplemented by horticultural LED lighting in a greenhouse at the Biosciences East and Central Africa (International Livestock Research Institute Hub, Nairobi Kenya).

Cassava plants at the 2–3 leaf stage were inoculated with infectious clones^39,40^ corresponding to ACMV DNA-A (MT858793.1) and DNA-B (MT858794.1) or to EACMCV DNA-A (MT856195) and DNA-B (MT856192) to obtain singly infected plants. All four plasmids were co-precipitated onto gold particles and co-inoculated to obtain co-infected plants. Inoculations were conducted using low pressure biolistic bombardment to deliver the plasmid DNAs^18^. Only symptomatic plants with infections confirmed by qPCR of viral DNA were used as sources for virus acquisition by whiteflies.

### Virus transmission

Plants infected with ACMV or EACMCV or co-infected with both viruses that differed in their respective DNA-A:DNA-B ratios were used as sources for whitefly transmission. DNA-A and DNA-B titers of each virus in the symptomatic plants were quantified by qPCR at the time the experiments were initiated. For virus acquisition by whiteflies, single plants of each virus treatment (ACMV only, EACMCV only or ACMV + EACMCV) were placed in separate whitefly-proof cages containing 400–500 one-to-five-day-old adult whiteflies. The acquisition access period (AAP) was 48 h. Whiteflies were then aspirated individually into 1.5-mL microcentrifuge tubes (one/tube) containing either a cassava leaf disc or a sucrose diet as the inoculation substrate and held in the dark for a 48-h inoculation access period (IAP). The leaf discs were cut from uninfected plants using a cork borer (1-cm diameter). The sucrose diet consisted of 63 µL of a 15% aqueous sucrose solution sealed within the “cup” formed by the inner surface of the microcentrifuge cap and covered using stretched Parafilm M® through which the whiteflies could feed. During both the AAP and IAP, whiteflies were maintained at 25 °C in the dark.

Upon completion of the IAP, all dead whiteflies and their associated inoculation substrates were discarded. Microcentrifuge tubes containing live whiteflies were chilled and the whiteflies placed in 95% ethanol. The whiteflies and the sucrose diets were then stored at − 20 °C. The leaf discs were held for an additional 48 h to allow virus replication before placing them at − 20 °C. Each whitefly and its corresponding inoculation substrate were labeled to allow data to be paired in subsequent analyses. Samples were maintained at − 20 °C until the DNA was extracted, and qPCR was performed to determine the titers of DNA-A and DNA-B of each virus.

### Plant tissue sampling and DNA extraction of plants and whiteflies

Leaf samples were taken from the plants used for virus acquisition by whiteflies to confirm infection and measure DNA-A and DNA-B titers prior to the start of the AAP. These samples consisted of one 0.5-cm diameter leaf disc cut from the basal region of the first, second and third fully expanded leaves below the plant apex. The sampled leaf positions represented sites where feeding whiteflies most frequently aggregated. Measurements of DNA-A and DNA-B titers acquired by whiteflies or transmitted by whiteflies to sucrose sachets or leaf discs were made on individual whiteflies, sucrose sachets and leaf discs.

Each leaf sample was placed in a 1.5-mL cryotube containing two 4.5-mm metal beads, flash frozen in liquid nitrogen and stored at − 20 °C. The samples were ground to a fine powder using a SPEX™ Geno Grinder 2010® (SPEX*SamplePrep, Metuchen, NJ) set at 1200 strokes/min for 5–10 s. DNA was extracted following the manufacturer's protocol in the Qiagen DNeasy Plant Mini Kit (Qiagen, Hilden, Germany) except that 50-uL of Buffer AE was passed through the column twice to maximize the amount of DNA extracted.

A single whitefly was placed to a 1. 5-mL cryotube filled ¼ of the way with 1.4-mm acid washed zirconium beads (OPS Diagnostics, Lebanon NJ), flash frozen with liquid nitrogen, and stored at − 20 °C. Insect tissue was homogenized as described for plant tissue. Total DNA was extracted from each whitefly using the Qiagen® DNeasy Blood and Tissue Kit (Qiagen, Hilden, Germany) following the manufacturer’s protocol, except that 100 uL of Buffer AL, 250 uL of AW1, 250 uL of AW2, and 50 uL of milli-Q water were passed through the membrane twice. Total DNA from leaf disks and whiteflies was measured using a Nanodrop 2000 Spectrophotometer® (Thermo Fisher Scientific Inc.) following manufacturers’ protocols for dsDNA.

### Copy number (titer) of viral DNA segments

Quantitative PCR (qPCR) was used to measure the copy number of the DNA-A and DNA-B genome segments of ACMV and EACMCV using the primer pairs and conditions described by Aimone et al.^18^ with the following modifications. The amplification reactions were performed in 20 µL consisting of a master mix with 10 µL of Maxima SYBR Green/ROX qPCR Master Mix (ThermoFisher Scientific), 0.5 µL (10 µM) of each primer and 4 µL of ultrapure water added to 5 µL of the sample (40 ng total leaf DNA, 3 ng total whitefly DNA, or 5 µL sucrose diet). The reactions were performed for 40 cycles in 96-well plates in an Applied Biosystems 7500 Real Time PCR Machine® (ThermoFisher Scientific) using the following reaction conditions: initial denaturation at 95 °C for 10 min, 40 cycles of 1 min at 60 °C (annealing + elongation) and 1 min at 95 °C (denaturation), and final incubation of 10 min at 95 °C. Three technical replicates/sample were included on each plate. Each qPCR plate also contained a standard curve (serial dilution of 3 × 10^7^ to 30 copies of a plasmid DNA containing the viral DNA segment), water control, and negative controls consisting of leaf disk and sucrose sachet samples that had been fed on by whiteflies that were not exposed to an infected source plant. Ct values were calculated using the Applied Biosystems 7500 Real Time PCR Machine® software. Segment copy number was calculated as described by Aimone et al*.*^18^ Samples were scored as positive if the copy number was greater than the mean plus 3 standard deviations of the negative controls and was within the range of the standard curve.

### Data analysis

Data on the titers of DNA-A and DNA-B segments in plants, whiteflies, sucrose, and leaf discs were transformed to log_10_ copy number per ng total DNA prior to statistical analysis. Ratios of DNA-A:DNA-B log_10_ copy number were calculated for each whitefly and transmission substrate (sucrose diet, leaf disc). All analyses treated individual whiteflies and substrates as the observational unit and data for individual whiteflies and transmission substrates were paired for analyses. All analyses were conducted in SAS version 9.4 (Cary, NC).

### Virus acquisition

Each whitefly that survived through the IAP was scored as having acquired or failing to acquire DNA-A or DNA-B or both DNA-A and DNA-B of each virus. Logistic regression was conducted using the GLIMMIX procedure in SAS to compare differences in the probability of a whitefly acquiring DNA-A and DNA-B. For whiteflies given an AAP on each virus (ACMC or EACMCV) and plant type combination (single infection or co-infection), two analyses were performed. The first compared probabilities of a whitefly acquiring DNA-A and DNA-B regardless of whether the cognate component was acquired, and the second compared the probabilities of acquiring DNA-A only, DNA-B only, or both DNA-A and DNA-B. The numbers of whiteflies included in these analyses differed among virus and infected plant combinations due to differences in whitefly survival.

To compare the DNA-A:DNA-B titer ratios acquired by whiteflies to the ratio in the infected plant on which they fed, one-sample t-tests were used to test the hypothesis that the DNA-A:DNA-B ratio in the whitefly did not differ from that in the infected plant. Separate analyses were conducted for each virus and virus source plant (singly infected or co-infected). Only whiteflies that acquired both segments of the virus were included in the analysis, but the ratios in whiteflies acquiring a given virus from the co-infected plant included individuals acquiring only both segments of one virus and those that also acquired at least one component of the heterologous virus.

For each virus, linear regression analyses were conducted using the GLM procedure in SAS to describe the relationships between the DNA-A and DNA-B log_10_ titers in whiteflies. Separate analyses were conducted for whiteflies that acquired both segments of only one virus regardless of whether the source plant was singly or co-infected, and for whiteflies that acquired both segments of one virus and at least one segment of the heterologous virus. To extend the range of segment titers included in these analyses, we included data from a preliminary experiment in which whiteflies had acquired lower levels of viral DNA. Analysis of variance and mean separation by LSMeans using the GLM procedure in SAS were conducted to test for significant differences in the DNA-A:DNA-B log_10_ titer ratios among whiteflies in the different virus and segment acquisition scenarios.

### Virus transmission

Logistic regression analyses were conducted to examine the effects of the titers of DNA-A and DNA-B acquired by a whitefly on the probability of their transmission to sucrose or leaf discs. An initial analysis revealed that co-acquisition of either or both heterologous viral segments by whiteflies had no effect on the probability of transmission of individual viral segments of either virus. Therefore, all whiteflies that had acquired a given segment were included in a subsequent analysis for that segment. Separate analyses were conducted for whiteflies transmitting to sucrose and leaf discs and for each segment of each virus. In these analyses, the probability of transmission was modeled as a function of the segment’s log_10_ titer in the whitefly. Paired t-tests were conducted using the same dataset to test the hypothesis that the DNA-A:DNA-B log_10_ titer ratios transmitted to sucrose or leaf discs did not differ from the corresponding ratios in the transmitting whitefly. One-sample t-tests were used to test the hypothesis that the DNA-A:DNA-B ratio in the whitefly did not differ from one.

### Research involving plants

The plant collection and use were in accordance with all the relevant guidelines.

## Supplementary Information

Supplementary Information.

## Data Availability

Data are available upon request from the corresponding author.
