# Supplementary material for: Genome segment ratios change during whitefly transmission of two bipartite cassava mosaic begomoviruses
Source: Sci Rep. 2023 Jun 21;13:10059. doi: 10.1038/s41598-023-37278-8 (PMC10284885; doi:10.1038/s41598-023-37278-8)
Supplement: Supplementary file 1 — Supplementary Information. [file 41598_2023_37278_MOESM1_ESM.pdf]

Supplementary Information for

Genome Segment Ratios Change During Whitefly Transmission of Two Bipartite Cassava Mosaic Begomoviruses

George G. Kennedy, William Sharpee, Alana L. Jacobson, Mary Wambugu, Benard Mware, Linda Hanley-Bowdoin

Paste corresponding author name: **George G. Kennedy**[1]
Email: gkennedy@ncsu.edu

This PDF file includes:

Table S1

Supplemental Table S1

Table S1. Regression parameter estimates: Relationship of viral genomic segment titer (log_{10} copy number) in transmitting whiteflies and probability of transmission of that segment to sucrose sachets and leaf discs.

Viral segment	Effect	Estimate	St. Error	DF	t Value	Prob.>t
**Sucrose sachet**						
ACMV DNA-A	Intercept	-3.6312	1.2642	1	-2.87	0.2133
	Titer	1.5967	0.4131	70	3.85	0.0003
ACMV DNA-B	Intercept	-5.0888	2.1241	1	-2.4	0.2517
	Titer	2.5343	0.6515	69	3.89	0.0002
EACMCV DNA-A	Intercept	-3.4852	1.036	1	-3.36	0.1839
	Titer	2.1186	0.4453	82	4.76	<0.0001
EACMCV DNA-B	Intercept	-4.921	1.5553	1	-3.16	0.1949
	Titer	2.1737	0.5194	74	4.19	<0.0001
**Leaf disc**						
ACMV DNA-A	Intercept	-2.1668	0.864	1	-2.51	0.2416
	Titer	1.0922	0.3028	55	3.61	0.0007
ACMV DNA-B	Intercept	-3.2201	0.8253	1	-3.90	0.1597
	Titer	1.2335	0.3301	57	3.74	0.0004
EACMCV DNA-A	Intercept	-5.7085	2.9026	1	-1.97	0.2995
	Titer	2.2180	1.0588	38	2.09	0.0429
EACMCV DNA-B	Intercept	-3.2305	1.116	1	-2.89	0.2118
	Titer	1.5232	0.4868	49	3.13	0.0030
